# Long-term legume green manure residue incorporation is more beneficial to improving bacterial richness, soil quality and rice yield than mowing under double-rice cropping system in Dongting Lake Plain, China

**DOI:** 10.3389/fpls.2025.1603434

**Published:** 2025-06-03

**Authors:** Jiangwen Nie, Qingyun Xie, Yong Zhou, Feng He, Muhammad Yousaf, Bo Zhu, Zhangyong Liu

**Affiliations:** ^1^ Hubei Key Laboratory of Waterlogging Disaster and Agricultural Use of Wetland/Engineering Research Center of Ecology and Agricultural Use of Wetland, Ministry of Education, College of Agriculture, Yangtze University, Jingzhou, China; ^2^ College of Life Science and Technology, Hubei Engineering University, Xiaogan, China

**Keywords:** green manure, microbial community, soil fertility, legume, crop yields

## Abstract

**Introduction:**

Chinese milk vetch (vetch) is a promising winter cover crop that can reduce dependence on fertilizers and enhance crop productivity in southern China. However, its impact on soil microbial communities, soil quality, and rice yield, particularly when comparing the incorporation of green manure residue to mowing, remains insufficiently explored.

**Methods:**

To address this gap, an 12-year field experiment was conducted in the Dongting Lake Plain, comparing three vetch management strategies under double-rice cropping system: winter fallow (CK), vetch rotation with residue incorporation (CMI), and vetch rotation with residue mowing (CMR). The soil quality index (SQI) was calculated based on abiotic [e.g., soil organic carbon (SOC), total nitrogen (TN), nitrate nitrogen (NO_3_–N)] and biotic [e.g., bacterial abundance, abundance-based coverage (ACE), Chao 1] factors.

**Results and discussion:**

Results indicated that both the vetch management strategies significantly improved rice yield. Compared to CK, CMI and CMR enhanced early, late, and annual rice yields by 6.22%-10.77%, 7.50%-13.49%, and 7.03%-12.40%, respectively. Additionally, CMI improved soil redox potential, alkali-hydrolyzale nitrogen, ammonium nitrogen, and NO_3_–N, while CMR enhanced soil SOC, TN, NO_3_–N levels. Both CMI and CMR resulted in increases in the bacterial ACE index by 2.43%-2.53% and the Chao1 index by 0.92%-2.88% (P < 0.05). Furthermore, CMI reduced the Shannon index by 1.17% but increased the Simpson index by 19.35%, while CMR increased the Shannon index by 1.73% and elevated the Simpson index by 19.35% (P < 0.05). Principal component analysis indicated distinct bacterial community structures between CK and CMR. The dominant bacterial phyla included Proteobacteria, Chloroflexi, Nitrospirae, Acidobacteria, Bacteroidetes, and Actinobacteria. Notably, CMR exhibited lower relative abundances of Proteobacteria, Nitrospirae, and Acidobacteria compared to CMI. Compared to CK, CMI increased SQI by 6.92%, while CMR showed more modest improvements in soil quality. Moreover, a strong positive correlation between rice yield and SQI further confirmed the beneficial effect of vetch rotation on soil fertility. These findings underscore the potential of vetch rotation, particularly through vetch incorporation, to enhance soil quality and rice productivity, thereby offering valuable insights for sustainable agricultural practices.

## Highlights

- The impact long-term use of green manure crops on soil quality, bacterial, and rice yield were investigated.
- Chinese milk vetch incorporation and mowing both benefit bacterial richness;
- Chinese milk vetch incorporation rather than mowing enhanced soil quality;
- Chinese milk vetch incorporation and mowing both improved rice yield;

## 1 Introduction

The challenge of maintaining a high standard of living while increasing crop yields to feed a growing global population has emerged as a critical issue (Fischer et al., 2014). China, as the world’s leading rice producer, has 60% of it’s rice fields located in the southern region, which is vital for ensuring both national and global food security (Li et al., 2018). However, the prevalence of winter fallow in the region presents significant challenges, with more than 50% of farmland lying idle in some provinces (Zhai et al., 2012; Wang et al., 2024). Reports indicated that approximately 9.3 million hectares of winter fields in these areas are underutilized, with 72% of them readily available for development and use (Tan, 2010). On the one hand, winter fallow not only wastes valuable light and heat resources but also accelerates soil erosion due to soil exposure, leading to the depletion of organic matter and essential nutrients (Wang et al., 2020). Furthermore, idle winter fields contribute to year-round soil flooding, placing the soil in an anaerobic state. This condition prevents the mineralization and release of soil nutrients, ultimately diminishing the fertility and productivity of rice fields (Yin et al., 2024). As a result, there is a growing focus on winter planting as a potential solution to soil degradation (Fang et al., 2021).

Soil health is a multifaceted concept that encompasses its biological, physical, and chemical properties, reflecting its ongoing role as a dynamic living ecosystem (Wood and Bowman, 2021). The use of winter cover crops not only boosts rice yields but also contributes to soil carbon sequestration, thereby supporting sustainable rice farming and mitigating soil degradation (Li et al., 2016; Xie et al., 2016; Yao et al., 2025). For instance, legumes used as green manure biologically fix nitrogen (N), enriching soil N levels for subsequent crops (Thorup-Kristensen et al., 2003). The long-term benefits of manure application have been proven to sustainably improve the supply of nutrients needed for crop growth (Demelash et al., 2014; Cai et al., 2018b). Moreover, green manure has been shown to increase soil organic carbon (SOC) by 14%-24% and reduce the need for synthetic fertilizers by 25%-51% when compared to fallow systems (Yao et al., 2017). The incorporation of green manure significantly improves soil fertility, particularly in terms of organic matter, N, and potassium (K), while supporting ecosystem health (Amusan et al., 2011; Jayaraman et al., 2021). However, due to its high N content and low carbon-to-nitrogen (C/N) ratio, leguminous green manure decomposes rapidly. Peak N mineralization typically occurs 2 to 4 weeks after incorporation (Zhou et al., 2019), which may not coincide with rice’s peak N uptake period, as farmers generally incorporate green manure just prior to rice transplanting. In addition, a recent study suggests that the benefits of Chinese milk vetch (*Astragalus sinicus* L., vetch) on soil fertility and rice yield may decline after five consecutive years of high-speed fertilization (Chen et al., 2020). Despite these findings, few studies have compared the effects of green manure on soil quality between residue incorporation and mowing. Therefore, it is crucial to investigate how different management practices of cover crop residue impact rice yields through soil nutrient dynamics.

The soil microbiome plays a critical role in soil function and fertility, with its composition and activity shaped by agricultural practices (Dai et al., 2021; Yang et al., 2023a). The incorporating green manure boosts soil nutrient availability and enriches the microbiome, thereby promoting improved nutrient uptake by plants (Wang et al., 2025). For example, Zhang et al. (2017) found that long-term double-rice-green manure rotations significantly altered the microbial community in the rice rhizosphere, enriching beneficial bacteria such as Acinetobacter and Pseudomonas under green manure treatments. The changes in microbial community structure and function in soil are influenced by the physical and chemical properties of the soil (Li et al., 2024; Lyu et al., 2025). Abiotic factors, such as land use, nutrient availability, pH levels, and the rhizosphere, regulate the composition of microorganisms in the soil, which subsequently alters soil characteristics. Thus, given their sensitivity to changes in soil conditions, microbial community diversity and abundance serve as reliable indicators of soil quality (Li et al., 2024). However, research on the effect of legume crop rotations on soil microbial communities remains inconsistent (Tiemann et al., 2015; Qin et al., 2017), and few studies have integrated microbial changes into soil quality assessments, particularly within legume-cereal rotation systems (Zhou et al., 2020a).

In southern China, the six-month winter fallow period between late rice harvest in October and early rice transplanting in April allows farmers to plant cover crops. The practice of planting green manure in paddy soils during winter is a traditional practice in China. When legumes, such as Chinese milk vetch, are utilized as green manure, they not only fix atmospheric N but also generate substantial biomass. Historically, farmers in this region have encountered challenges due to a shortage of feed materials for their livestock, leading to the common practice of mowing most of the vetch straw for cattle feed at harvest (Haque et al., 2015; Nie et al., 2018). However, the effect of total green manure residue mowing compared to incorporation on soil microbial communities, soil quality, and rice yield remain inadequately explored. Therefore, we conducted an 12-year field experiment (2008-2020) to assess the effects of green manure on soil microbial communities, soil quality and rice yield. The objective of this study was to examine the effects of different vetch managements strategies-namely, winter fallow, rotational cultivation with milk vetch incorporating residue, and rotational cultivation with vetch while employing residue mowing on soil properties, microbial composition, soil quality and rice yield. Our hypotheses were that: (1) rotation with vetch would increase soil nutrient concentrations (e.g., SOC and available nutrients) and improve overall quality; (2) rotation with vetch would enhance soil microbial richness and alter community structure; and (3) the incorporation of vetch into soil would be more effective in improving soil quality and rice yield than mowing.

## 2 Materials and methods

### 2.1 Experimental site

The field experiment commenced in March 2008 in Huarong County, situated in Dongting Lake, Hunan Province, China (29°52′N, 112°55′E). This region experiences a humid subtropical monsoon climate, characterized by a mean annual air temperature ranging from 16.0 to 18.0°C and average annual precipitation between 1200 and 1700 mm. During the early rice growing period, the annual temperature was recorded at 23.0°C, with an accumulated temperature (≥10°C) of 2208°C, an average precipitation of 502 mm, and average sunshine duration of 456 hours. In contrast, the late rice growing period exhibited annual temperatures of 17.5°C, accumulative temperatures of 2433°C, average precipitation of 440 mm, and average sunshine duration of 722.4 hours. The experimental soil was identified as a purple calcareous clayey paddy soil, formed from sediment deposited by the Yangtze River. Prior to the experiment, the chemical properties of the topsoil (0–20 cm) were assessed, revealing the following values: soil pH (H_2_O) of 7.10, soil organic carbon (SOC) of 28.54 g kg^−1^, total nitrogen (TN) of 3.11 g kg^−1^, available phosphorus (AP) of 16.40 mg kg^−1^, and available potassium (AK) of 69.00 mg kg^−1^.

### 2.2 Experimental design

**Figure 1:**
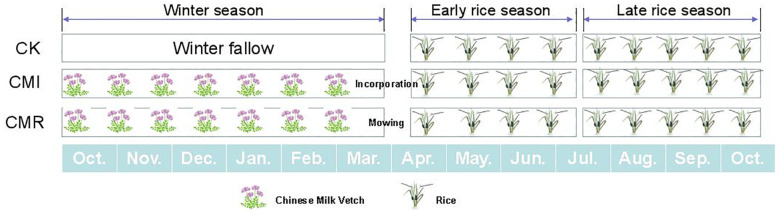
The picture illustrates the three fertilization regimes: winter fallow (CK), rotation with and incorporation of Chinese milk vetch (CMI), and rotation with and mowing of Chinese milk vetch (CMR) within a double-rice cropping system in Southern China. The winter fallow in double-rice cropping system represents the traditional local cropping system in this context. The width of each crop picture corresponds to the relative duration of its growth period.

The experimental treatments comprised three fertilization regimes (**Figure 1**): (1) winter fallow within a double-rice cropping system (CK); (2) rotation with and incorporation of Chinese milk vetch in the double-rice system (CMI); and (3) rotation with and mowing of Chinese milk vetch in the double-rice system (CMR). Each treatment was replicated in three plots (3.3 m × 9 m), which were arranged randomly and separated by concrete furrows. The rice varieties used were early rice “Zhefu No. 7” and late rice “Longxiangyou 130” (*Oryza sativa* L.), while the green manure employed was Chinese milk vetch, specifically “Xinagfei No. 3.” The vetch was incorporated into the soil at full bloom, about two weeks before early rice transplanting. Fertilizer application and field management practices adhered to local farmer conventions (Zhu et al., 2016; Nie et al., 2019).

### 2.3 Plant and soil sampling and biochemical properties

At the late rice harvest (Mid-October 2020), we select diagonally two 1-m² areas to take sample from each subplot to measure rice grain yield (12.5% moisture content). Soil samples were collected from the topsoil (5–20 cm) at five random locations within each plot using a 20 mm diameter soil auger. After removing stones, sand, and plant debris, the samples from these points were combined, sealed in bags, and stored. Tiny roots and remaining debris were removed using a 2-mm sieve, and all samples were preservation at 4°C. One set of samples was preservation at −80°C for microbial community analysis, while the other was air-dried in the laboratory for chemical analysis.

Soil pH was determined using a pH meter with a soil-to-water ratio of 1:2.5 and soil redox potential (Eh) was measured directly by molybdenum electrodesoil Eh (Lu, 2000). Ammonium nitrogen (NH_4_^+^-N) levels were determined through 2 mol L^−1^ KCl extraction and indigo blue colorimetry, while nitrate nitrogen (NO_3_^–^N) was quantified via double-wavelength ultraviolet spectrophotometry. TN and total carbon (TC) were analyzed using an element analyser (Costech ECS 4010, CHNSO, Germany). The C/N ratio was calculated based on the masses of total C and N. SOC was measured using the K_2_Cr_2_O_7_ redox titration method (Bao, 2000), and alkali-hydrolyzed nitrogen (AN) was determined through alkaline hydrolysis (Bao, 2000).

### 2.4 DNA Extraction, PCR amplification, and Illumina MiSeq sequencing

A 0.5 g soil sample was subjected to DNA isolation using the FastDNA^®^ SPIN Kit for Soil (QBIOgene, USA). For the purpose of performing PCR amplification, primers specific to the V3-V4 region of the 16S rRNA gene were utilised: 338F and 806R (Walter et al., 2000). The PCR reactions was constituted of 25 μL of 2× Premix Taq (TaKaRa Biotechnology, Dalian Co. Ltd., China), 1 μL of each primer (10 pmol mL^-1^), and 3 μL of DNA template (20 ng μL^-1^), resulting in a total of volume of 50 µL. The amplification was performed using a thermocycling protocal that included an initial denaturation step at 94°C for 5 min, followed by 30 cycles of denaturation at 94°C for 30 s, annealing at 52°C for 30 s, and extension at 72°C for 30 s. The procedure concluded with a 10-minute elongation step at 72°C. Subsequent to this, the PCR products were then pooled in equal amounts, purified, and sequenced using the Illumina MiSeq platform.

### 2.5 Real-Time PCR

Real-time PCR amplification of the bacterial 16S rDNA gene was measured using an ABI 7500 thermocycler (Applied Biosystems) with SYBR^®^ Premix Ex TaqTM (Takara), following the manufacturer’s instructions. Each 25 µL reaction contained 1× SYBR Premix Ex Taq, 10 µM of each primer (1369F and 1541R) (Suzuki et al., 2000), and 1 µL of DNA template. The amplification protocol included initial denaturation at 95°C for 2 minutes, followed by 40 cycles of 95°C for 10 seconds, 60°C for 1 minute, and data collection at the end of each cycle. Ct values were recorded using 7500 System SDS software.

A standard curve for 16S rDNA was constructed by cloning the PCR product into the pMD18-T vector (Shen et al., 2010), extracting and sequencing the plasmids, and preparing a 10-fold dilution series (10^8^ to 10³ copies). The amplification efficiency was 94.4%, with an R² value of 0.999.

### 2.6 Processing of sequencing data

The raw FASTQ data were demultiplexed and subjected to quality filtering using QIIME (version 1.17), in accordance with methodologies outlined in prior research (Yang et al., 2018). The alpha diversity of bacterial communities were then assessed using diversity indices (Shannon and inverse Simpson) and richness estimators [abundance-based coverage (ACE) and Chao1] computed with MOTHUR software (version 1.30.1) (Zhou et al., 2015; Xun et al., 2016).

### 2.7 Soil quality index

The evaluation of the SQI involved the normalisation of each soil indicator to a value ranging from 0 to 1, according to the following Equation (1) (Zeraatpisheh et al., 2020; Zhou et al., 2021a):


(1) $$S_{i}=\frac{X_{i}}{X_{\text{max}}}$$

where Si denotes the linear score for parameter i, with a range from 0 to 1; X represents the observed value; X_max_ indicates the maximum mean value for parameter i; and X_min_ refers to the minimum mean value of soil parameter i. Soil indicators were grouped into two categories according to their sensitivity to soil quality.

The subsequent step was to calculate the Soil Quality Index (SQI). To do so, the SQI-area method was emplyed accroding to Equation (2), which involves the comparison of area on a radar plot that have been created from all the relevant soil parameters (Kuzyakov et al., 2020).


(2) $$SQI=0.5\times \sum_{i}^{n}S_{i}^{2}\times \sin(\frac{2\pi }{n})$$

where π is defined as 3.14, and n denotes the number of indicators used to calculate the SQI. The areas on the radar chart were derived from a combination of nine soil parameters: pH, Eh, SWS, NO_3_^–^N, NH_4_^+^-N, AN, TC, TN, C/N, as well as the 16S rDNA abundance, ACE, Chao 1, Shannon, and Simpson indices, which were calculated as the SQI-area in this study.

### 2.8 Statistical Analysis

Under the condition of a significance threshold of 0.05, one-way analysis of variance (ANOVA) is used to evaluate the impacts of different fertilization treatments on soil properties, bacterial abundance, and bacterial community α-diversity indices. IBM SPSS Statistics (version 20.0) is used for analysis, and the least significant difference (LSD) test (*P* = 0.05) is adopted. In addition, Pearson correlation analysis was also conducted to explore the relationships among soil characteristics, bacterial abundance and α diversity indices. At the same time, IBM SPSS Statistics (version 20.0) was used. In the statistical program R (version 3.2.1), principal component analysis (PCA) was performed with the help of the “vegan” software package (Team et al., 2014). The graphical representation was generated with the help of SigmaPlot 12.5 (Systat Software Inc., San Jose, California, USA).

## 3 Results

### 3.1 Soil properties

**Figure 2:**
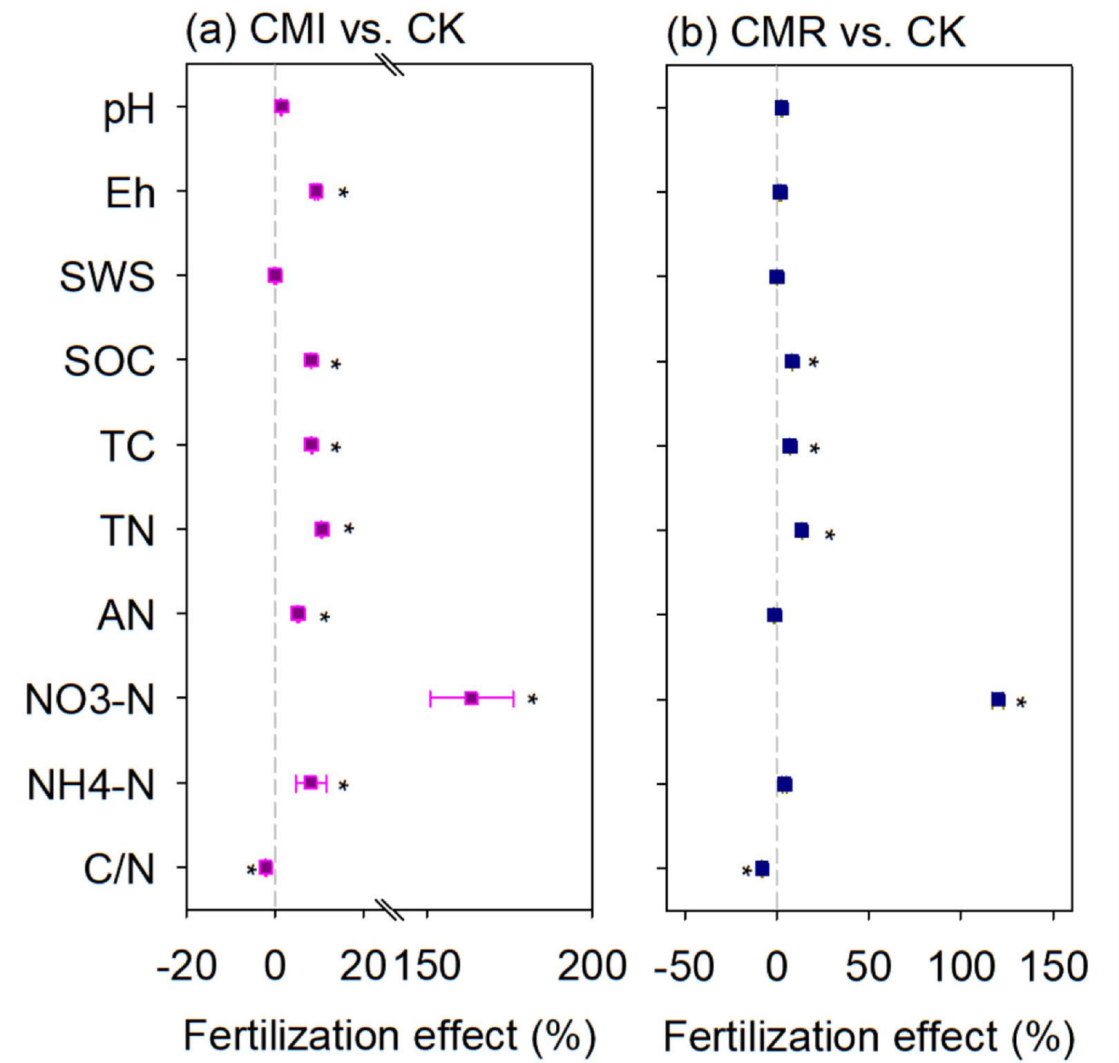
The fertilization effects (%) of vetch incorporation **(a)** and vetch mowing **(b)** on soil properties at a depth of 0–20 cm. The CK, CMI, and CMR represent winter fallow, rotation with and incorporation of Chinese milk vetch, and rotation with and mowing of Chinese milk vetch in a double-rice cropping system, respectively. The assessed soil properties include soil pH and Eh, soil water content (SWS), soil organic carbon (SOC) content, total nitrogen (TN) content, soil total carbon (TC) content, and their ratio (C/N); Alkali-hydrolyzed nitrogen (AN), soil ammonium nitrogen (NH_4_^+^-N), and nitrate nitrogen (NO_3_^–^N) content. The values presented are averages (with 95% CI) from three replicates. *indicate statistically significant differences between treatments (*p* < 0.05).

In comparison to CK, the CMI treatment significantly enhanced several soil properties: soil Eh increased by 9.29%, TC by 8.34%, SOC by 8.21%, TN by 10.52%, NH_4_^+^-N by 8.07%, and NO_3_^–^N by an impressive 163.59% (**Figure 2a**, *P* < 0.05). Conversely, the CMR treatment resulted in increases in TC by 4.52%, SOC by 4.03%, TN by 13.52%, and NO_3_^–^N by 120.11%. Notably, the CRI treatment led to a decrease in the C/N by 2.08%, while the CRM treatment caused a more significant reduction of 7.79% in C/N when compared to CK (**Figure 2b**).

### 3.2 Bacterial abundance and alpha diversity

**Figure 3:**
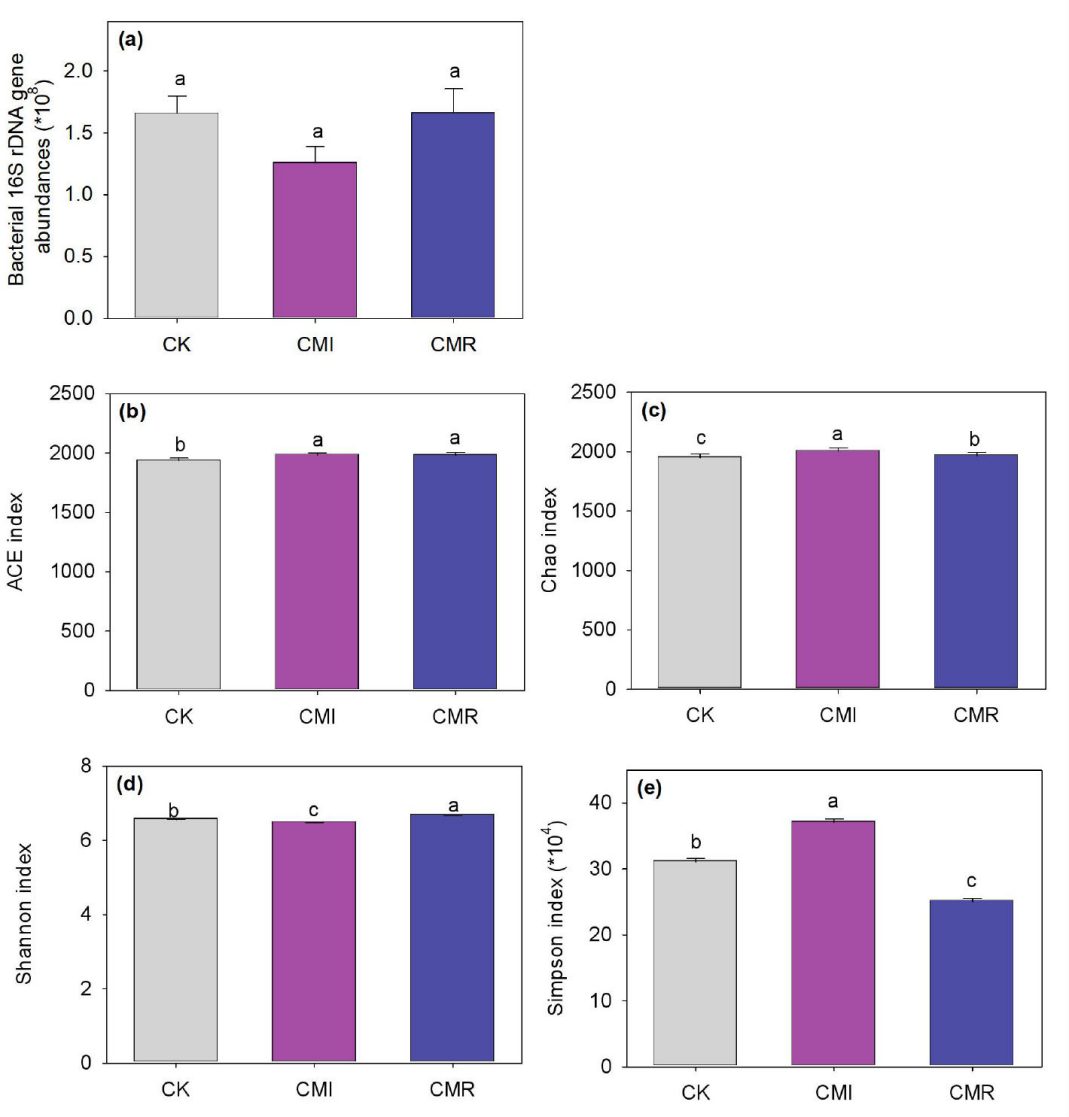
Bacterial 16S rDNA gene abundance **(a)** and alpha diversity **(b-e)** of communities response to different fertilization. The CK, CMI, and CMR represent winter fallow, rotation with and incorporation of Chinese milk vetch, and rotation with and mowing of Chinese milk vetch in a double-rice cropping system, respectively. Bars labeled with different lowercase letters indicate significant differences among different fertilization (*p* < 0.05). Values presented are the average (± SE) of three replicates. *means multiply by multiple. .

No significant differences in bacterial abundance were observed among the three treatments (**Figure 3a**). However, when compared to CK, both CMI and CMR treatments resulted in increases in the bacterial ACE index by 2.43% to 2.53% and the Chao 1 index by 0.92% to 2.88% (*P* < 0.05, **Figures 3b, c**). Additionally, the CMI treatment decreased the Shannon index by 1.17% while increasing the Simpson index by 19.35%. In contrast, the CMR treatment increased the Shannon index by 1.737% and also raised the Simpson index by 19.35% (*P* < 0.05) when compared to CK (**Figures 3d, e**).

### 3.3 Bacterial beta diversity, community compositions and its relationship with soil properties

**Figure 4:**
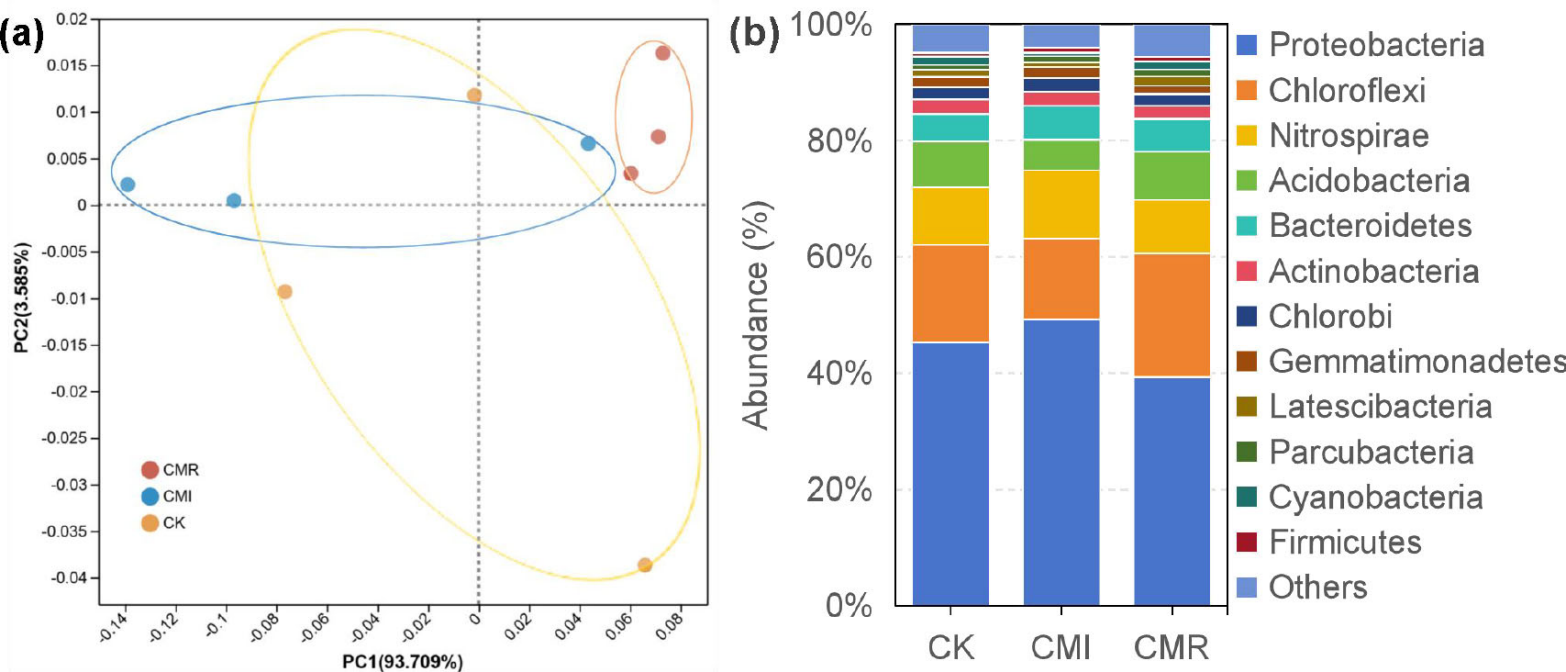
Principal component analysis [PCA, **(a)**] at the operational taxonomic unit (OTU) level and relative abundance (%) of the bacterial **(b)** abundant phylums in the rhizosphere soils under three fertilization regimes. The phyla considered abundant were those exhibiting an average proportion exceeding 1% across all treatments. The CK, CMI, and CMR represent winter fallow, rotation with and incorporation of Chinese milk vetch, and rotation with and mowing of Chinese milk vetch in a double-rice cropping system, respectively. Bars labeled with different letters indicate significant differences between different fertilization (*p* < 0.05). Values presented are the average (± SE) of three replicates. .

The structure of the soil bacterial community across different treatments was analyzed using Principal Component Analysis (PCA) (**Figure 4a**). The first two principal components, PC1 and PC2, explained 93.71% and 3.59% of the total variation, respectively. The CMI and CK treatments were closely grouped together, while a distinct separation was observed from CMR (ADONIS R = 0.649, *P* = 0.03).

The major bacterial phyla found in all samples included Proteobacteria (39.35%-49.32%), Chloroflexi (13.80%-21.29%), Nitrospirae (9.18%-11.75%), Acidobacteria (5.25%-8.27%), Bacteroidetes (4.66%-5.85%), and Actinobacteria (2.22%-2.51%) (**Figure 4b**). Notably, the relative abundance of Proteobacteria, Nitrospirae, Acidobacteria, and Gemmatimonadetes was higher in the CMI treatment compared to CMR, while Chloroflexi and Latescibacteria were more abundant in CMR than in CK (**Figure 4b**). In addition, Bacteroidetes showed a higher relative abundance in CMI than in CK.

**Figure 5:**
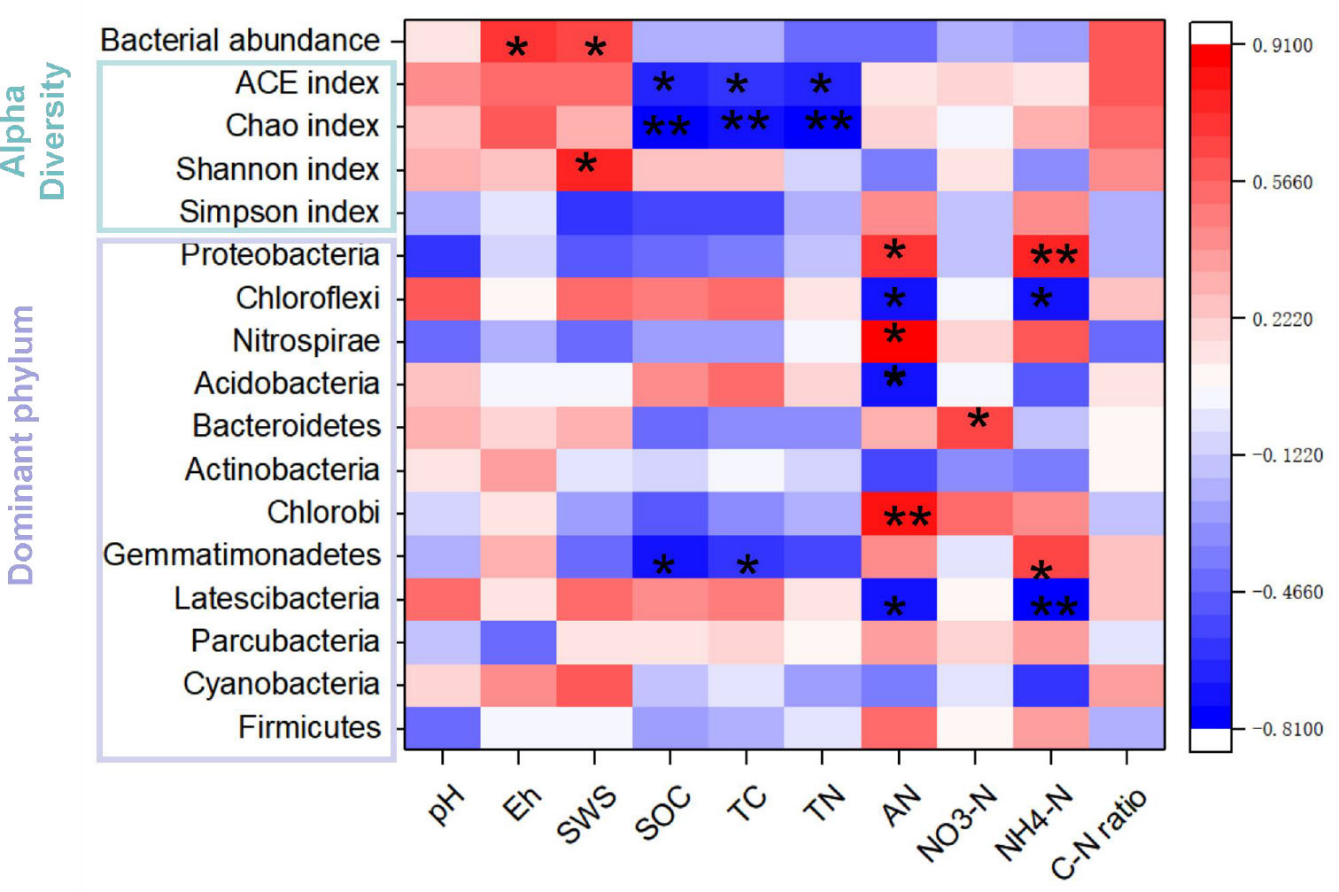
Correlation heatmap reveals the relationships between soil properties and bacterial gene abundance, the alpha diversity index and the predominant phyla in the rhizosphere soils under three fertilization regimes. The CK, CMI, and CMR represent the winter fallow, the rotation with and incorporation of Chinese milk vetch, and the rotation with and mowing of Chinese milk vetch within a double-rice cropping system, respectively. *, ** indicate *p* < 0.05 and *p* < 0.01, respectively.

Pearson correlation analysis (**Figure 5**) showed a positive correlation between bacterial abundance and soil Eh, as well as soil water saturation (SWS) (*P* < 0.05). Both the ACE and Chao indices exhibited negative correlations with SOC, TC, and TN, while the bacterial Shannon index was positively correlated with SWS. Additionally, soil AN showed positive correlations with Proteobacteria, Nitrospirae, and Chlorobi, but negative correlations with Chloroflexi, Acidobacteria, and Latescibacteria. Furthermore, soil NH_4_^+^-N had a positive correlation with Proteobacteria and Gemmatimonadetes, but a negative one with Chloroflexi and Latescibacteria. Lastly, soil NO_3_^–^N was positively correlated with Bacteroidetes, and both SOC and TC had positive correlations with Gemmatimonadetes.

### 3.4 Soil quality index and rice grain yields

**Figure 6:**
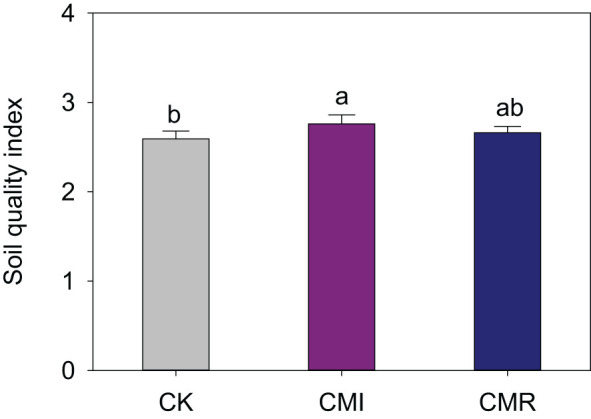
Soil quality index (SQI) responds to different fertilization regimes. The CK, CMI, and CMR represent winter fallow, rotation with and incorporation of Chinese milk vetch, and rotation with and mowing of Chinese milk vetch in a double-rice cropping system, respectively. Bars with different lowercase letters indicate significant differences between different fertilization (*p* < 0.05). Values presented are the average (± SE) of three replicates.

**Figure 7:**
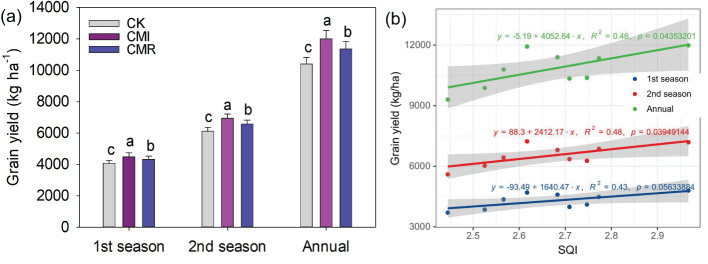
Rice grain yield **(a)** response to different fertilization and its relationship with SQI **(b)**. The CK, CMI, and CMR represent winter fallow, rotation with and incorporation of Chinese milk vetch, and rotation with and mowing of Chinese milk vetch in a double-rice cropping system, respectively. Bars with different lowercase letters indicate significant differences between different fertilization (*p* < 0.05). Values presented are the average (± SE) of three replicates.

In comparison to CK, the incorporation of Chinese milk vetch in crop rotation significantly enhanced the Soil Quality Index (SQI), particularly for CMI, which exhibited an improvement of 6.92% (*P* < 0.05, **Figure 6**). Furthermore, rotation with Chinese milk vetch led to an increase in rice grain yield. Specifically, the CMI resulted in enhancements of 10.77%, 13.49%, and 12.4% in early, late, and annual rice grain yields, respectively. In contrast, CMR yielded increases of 6.22%, 7.5%, and 7.03% for early, late, and annual rice grain yields when compared to CK (**Figure 7a**). Notably, a significant positive correlation was identified between the SQI and rice yield, particularly for late rice (R² = 0.48, *P* = 0.039; **Figure 7b**) and for annual rice yield (R² = 0.46, *P* = 0.046; **Figure 7b**).

## 4 Discussion

### 4.1 Influence of vetch on soil quality

Previous studies have proved that the amendment of green manure in agro-ecosystems is an effective strategy for enhancing soil fertility (Xie et al., 2016; Yang et al., 2019; Zhou et al., 2020b). For example, one study confirmed that an increasing the application of green manure amendment leads to higher level of soil organic carbon and nutrients, thereby improving soil quality (Wang et al., 2025). Similarly, our research indicates that planting milk vetch can significantly enhance soil quality upon its return to the field. This improvement is primarily attributed to the substantial of green manure straw, which effectively elevates the soil’s C and N content, particularly in terms of SOC, TN and AN nutrients (**Figure 2**, Hu et al., 2023; Wang et al., 2025; Daba et al., 2025). Furthermore, our findings suggest that long-term incorporation of organic matter, as compared to mowing, is more effective in increasing soil C and N content. Other studies have similarly indicated that the input amount of organic matter plays a crucial role in enhancing soil carbon level (Haque et al., 2015). However, it is critical to note that during the decomposition process of green fertilizer returned to the field, a significant amount of oxygen is consumed, leading to a transformation of oxidizing organic acids in the soil into reducing organic acids (Chen et al., 2016). Consequently, the total amount of reducing substances in the soil increases significantly (Xiao et al., 2013), resulting in a decrease in soil Eh. Contrary to previous studies, our research found that continuous input of organic matter through CMI significantly increased soil Eh. This increase may be attributed to the incorporation of vetch, which enhances soil structure (including improved soil aggregate structure, Yang et al., 2012; Hu et al., 2023), thereby improving soil aeration, increasing soil oxygen content, alleviating the accumulation of reducing substances, and ultimately leading to the increase in soil Eh.

In the rice rotation system, the growth process and incorporation of the rice crop significantly alter the N status of the soil in the paddy field, subsequently affecting the N conversion processes (Daba et al., 2025). Studies indicates that approximately 78% of N during the full flowering period is derived from biological N fixation, with about 93 kg hm^-2^ being added to the soil annually through the incorporation of above-ground and root residues (Cai et al., 2018a). Furthermore, 25%-45% of N in rice is sourced from the incorporation of legume green fertilizer (Singh et al., 2005). Consequently, there is a notable increase in soil N levels, including TN and NO_3_^–^N. Additionally, when compared with CMI, CMR has a more pronounced effect on reducing the of soil C/N ratio, primarily due to a lesser increase in soil C. This observation aligns with prior studies, which suggest that the low C/N ratio of leguminous crops results in a higher decomposition rate of leguminous straw, facilitating the rapid release of nutrients, particularly N, thereby influencing soil C/N (Virk et al., 2021). Overall, our results indicate that incorporation or mowing can effectively enhance soil quality through the increase of soil C and N nutrients.

### 4.2 Influence of vetch on soil bacterial community

Studies showed that, compared to the application of inorganic fertilizers alone, the combined use of vetch significantly enhanced both the total quantity and diversity of bacteria (Zhang et al., 2017; Wang et al., 2025). Our study corroborates these findings, indicating that the return of vetch straw to the field, irrespective of its initial presence, significantly increases bacteria richness. This enhancement is mainly attributed to elevated soil nutrient levels, particularly C and N, which stimulate microbial activity and consequently boost bacteria richness (Zhang et al., 2017; Wang et al., 2025). Furthermore, our results indicate a significant positive correlation between bacterial abundance and soil C and N content (**Figure 5**). However, we observed that incorporation of vetch to the field resulted in a decrease in bacterial diversity (shannon index), while a mowing strategy increased bacterial diversity. This suggests that the management strategy (regarding the amount of green fertilizer returned to the field) has a substantial impact on soil bacterial diversity. Similarly, Lyu et al. (2025) found that the use of total green manure resulted in a decrease in bacterial alpha diversity. Their research highlighted soil pH and mineral nitrogen content (NH_4_^+^-N and NO_3_^−^-N) as key factors driving this reduction in bacterial diversity. Additionally, other studies have suggested that soil bacteria are highly responsive to environmental fluctuations, with changes in soil pH and N availability having a substantial impact on the composition of microbial communities (Richardson et al., 2009; Yang et al., 2023b). Furthermore, long-term application of winter green fertilizers has been shown to modify the microbial food web structure in soil, increasing the fungi-to-bacteria ratio and shifting the microbial community toward a fungal-dominated composition (Chen et al., 2019). Overall, the application of large or excessive amounts of organic fertilizer over extended periods can lead to soil nutrient enrichment, which in turn affects the life history strategies of specific microbial groups (Li et al., 2021), such as eutrophic and oligotrophic bacteria, ultimately influencing microbial alpha diversity.

Previous studies have revealed that the main bacterial phyla present in soil include Proteobacteria, Actinobacteria, Acidobacteria, and Chloroflexi, which together represent approximately 80% of the total bacterial community (Lyu et al., 2025). Our research corroborates these findings, revealing that the dominant bacterial phyla also include Nitrospirae and Bacteroidetes. Furthermore, it has been established that different treatments of milk vetch significantly alter bacterial community structure. For instance, Lyu et al. (2025) reported a notable increase in Actinobacteria under the treatment of milk vetch returned to the field, identifying this community as beneficial for soil health. Similarly, our study indicated that CMR significantly influenced bacterial community structure. Specifically, when compared to CMI, soil treated with CMR demonstrated notable difference; CMI exhibited higher relative abundances of Proteobacteria, Nitrospirae, and Acidobacteria than CMR. This change can be interpreted as the complete integration of green plant fertilizers, resulting in actinomycetes becoming the dominant bacterial group in the soil. Actinomycetes typically thrive in alkaline soils, as they are able to secrete a large amount of extracellular hydrolytic enzymes that degrade various refractory organic components (Sanford, 2006; Gao et al., 2021). It is important to note that the symbiotic relationship between the rhizobia of woody plants and microorganisms such as actinomycetes stimulates the decomposition of cell wall exudates produced by other microorganisms (Li et al., 2019). This process usually leads to the accumulation of a large amount of organic acids and other secretions from the rootstock. These substances play a key role in the connection of solutes, nutrients and minerals (Van Zwieten et al., 2014). This interaction creates the rhizosphere environment and provides the culture with a higher ability to obtain food substances and a more stable structural foundation (Lyu et al., 2025). Continuous research shows that there is a close relationship between the microbial community and sulfonamide parameters (**Figure 5**), which means that including sulfonamide will affect the acquisition of nitrogen and the bacterial community structure compared to the removal of Aster. Overall, our study illustrates the effects of total vetch residue incorporation under rotation on soil bacterial diversity and structure, particularly beneficial microorganisms, thereby contributing to enhanced soil biological fertility.

### 4.3 Influence of vetch on rice yield

Our study found that winter legume cropping generally increased rice grain yield, aligning with previous research (Yang et al., 2013; Song et al., 2024). Notably, CMI treatment consistently resulted in the highest early, late, and overall rice yields when compared to CMR. This suggests that incorporating milk vetch into the soil is more beneficial for nutrient accumulation, including increases in soil carbon and nitrogen (Daba et al., 2025). The lack of vetch residue return during winter may have intensified nutrient competition between microorganisms and early rice. Additionally, the vetch rotation model promotes significant rhizodeposition, along with root senescence and decomposition over the winter (Virk et al., 2021), which allows for the retention of excess nutrients in the soil, making them available for early rice absorption, ultimately enhancing early rice yields (Pround et al., 2023). By returning straw, the nutrients absorbed by early rice straw were further increased, and the annual grain yield of rice was improved (Zhou et al., 2021b). Previous studies also showed that there was a close relationship between the yield of rice and the nutrient level of soil (Yang et al., 2013). Winter planting can improve soil fertility and increase the yield of rice (Yang et al., 2013; Song et al., 2024). During the decomposition process of this compound, the slow release of nutrients helps maintain the long-term fertility of the soil and improve the efficiency of nutrient utilization during rice transplantation (Meng et al., 2019). In contrast, the addition of vetch straw may lead to nutrient competition because part of the nitrogen is fixed by microorganisms and converted into microbial biomass, which is not available to the culture in the short term (Kuzyakov and Xu, 2013; Wang et al., 2018). This phenomenon may explain the relatively insignificant relationship between soil quality and early rice yield. Overall, our study indicates that long-term winter planting of milk vetch can significantly increase the yield of double-cropping rice, regardless of whether the green manure straw is returned to the field.

Given the consistent yield improvements observed with CMI, we recommend incorporating milk vetch directly into the soil during winter to maximize the nutrient benefits for double-cropping rice systems. However, it is important to consider local environmental factors such as climate and soil type, particularly in regions like the Dongting Lake Plain, where seasonal and geographical variations in temperature, precipitation, and soil properties may influence the growth of vetch and its impact on soil quality. In these areas, a more tailored approach may be necessary to optimize the benefits of milk vetch cultivation. Further field studies across different years and regions are recommended to refine these findings and establish best practices for sustainable rice production.

## 5 Conclusion

In conclusion, the 12-year field experiment conducted in the Dongting Lake Plain demonstrates that vetch rotation, particularly with residue incorporation, significantly enhances rice yield and soil quality compared to winter fallow. Both vetch management strategies (CMI and CMR) improved rice yields by up to 12.4% and positively influenced soil microbial diversity, with CMI showing a more pronounced improvement in soil redox potential and N availability. The study also highlights the beneficial effects of vetch rotation on soil quality, as reflected in the increase in the SQI and a strong correlation between SQI and rice yield. These findings support the potential of vetch rotation, particularly residue incorporation, as an effective sustainable agricultural practice to enhance both soil fertility and crop productivity in southern China.

## Data Availability

The original contributions presented in the study are publicly available. This data can be found here:NCBI, PRJNA1267802.
